# Menopausal symptoms and bone health in women undertaking risk reducing bilateral salpingo-oophorectomy: significant bone health issues in those not taking HRT

**DOI:** 10.1038/bjc.2011.202

**Published:** 2011-06-07

**Authors:** J Challberg, L Ashcroft, F Lalloo, B Eckersley, R Clayton, P Hopwood, P Selby, A Howell, D G Evans

**Affiliations:** 1 Department of Genetic Medicine, The University of Manchester, Manchester Academic Health Science Centre, Central Manchester Foundation Trust, St Mary's Hospital, 6th Floor, Oxford Road, Manchester M13 9WL, UK; 2 Manchester Breast Centre, University of Manchester, The Christie NHS Foundation Trust, Withington, Manchester M20 4BX, UK; 3 Breast Screening Service, Genesis Breast Cancer Prevention Centre, University Hospital of South Manchester NHS Trust, Wythenshawe, Manchester M23 9LT, UK; 4 Department of Medicine, The University of Manchester, Manchester Academic Health Science Centre, Central Manchester Foundation Trust, Oxford Road, Manchester M13 9WL, UK; 5 Department of Psychonchology, Institute of Cancer Research, Downs Road, Sutton, Surrey, UK

**Keywords:** oophorectomy, HRT, osteoporosis, osteopenia, menopause, breast cancer

## Abstract

**Background:**

Women at high ovarian cancer risk, especially those with mutations in BRCA1/BRCA2, are encouraged to undergo bilateral risk-reducing salpingo-oophorectomy (BRRSPO) prior to the natural menopause. The decision to use HRT to cover the period of oestrogen deprivation up to 50 years of age is difficult because of balancing the considerations of breast cancer risk, bone and cardiovascular health.

**Methods:**

We reviewed by questionnaire 289 women after BRRSPO aged ⩽48 years because of high ovarian cancer risk; 212 (73%) of women responded.

**Results:**

Previous HRT users (*n*=67) had significantly worse endocrine symptom scores than 67 current users (*P*=0.006). A total of 123 (58%) of women had ⩾24 months of oestrogen deprivation <50 years with 78 (37%) never taking HRT. Bone density (DXA) evaluations were available on 119 (56%) women: bone loss with a T score of ⩽−1.0 was present in 5 out of 31 (16%) women with no period of oestrogen deprivation <50 years compared with 37 out of 78 (47%) of those with ⩾24 months of oestrogen deprivation (*P*=0.03).

**Interpretation:**

Women undergoing BRRSPO <50 years should be counselled concerning the risks/benefits of HRT, taking into consideration the benefits on symptoms, bone health and cardiovascular health, and that the risks of breast cancer from oestrogen-only HRT appear to be relatively small.

Ovarian cancer is the fifth most common cause of cancer mortality in Western women and causes more deaths than all other cancers of the reproductive organs combined (Morrison *et al*, 2002). Approximately 8–15% of ovarian cancers are thought to be caused by inheritance of germline mutations in high-risk cancer-predisposing genes, such as *BRCA1* and *BRCA2* (Schildkraut and Thompson, 1988; Whittemore *et al*, 1997; Risch *et al*, 2001; Antoniou *et al*, 2003). The risk of ovarian cancer by age 70 in *BRCA1* mutation carriers is between 39–63%, and 11–30% for *BRCA2* carriers (Antoniou *et al*, 2003; Evans *et al*, 2008). Approximately 75% of women present with stage III/IV cancer, and 5-year survival for women presenting at stage IV is <5% (Teneriello and Park, 1995). The efficacy of screening methods, which include pelvic examination, transvaginal ultrasound and serum CA125 analysis, remains to be proven (Oei *et al*, 2006; Hermsen *et al*, 2007; Evans *et al*, 2009a, 2009b). Bilateral risk-reducing salpingo-oophorectomy (BRRSPO) is, therefore, often undertaken, and has been shown to reduce the risk of ovarian cancer by 96% and breast cancer by 53% (Rebbeck *et al*, 2002).

While reducing cancer risk, BRRSPO is not without consequences, particularly those relating to oestrogen deprivation from surgically induced menopause. These include symptoms of oestrogen deprivation, including hot and cold flushes, gastrointestinal problems, alterations in mood and sexual dysfunction (Elit *et al*, 2001; Madalinska *et al*, 2005; Gallicchio *et al*, 2006). Oestrogen deprivation also leads to loss of bone mineral density (BMD) and risk of osteoporosis and bone fracture (Jones *et al*, 1985; Cummings *et al*, 1998), and may shorten lifespan related to earlier onset cardiovascular disease (Howell and Evans, 2011). The greatest rate of BMD loss occurs soon after oophorectomy, and is reported to be as high as 20% in the first 18 months (Cann *et al*, 1980). The rate of bone loss reduces with time, but early menopause is a risk factor for fractures much later in life (van der Voort *et al*, 2003).

Hormone replacement therapy reduces symptoms associated with menopause in some women, and protects against bone density loss. However, HRT, particularly combined oestrogen and progesterone preparations, increases breast cancer risk and use is relatively contraindicated in women with or at high risk of breast cancer, although, paradoxically, recent observational data indicate that HRT use does not increase risk of breast cancer after BRRSPO in *BRCA1* and possibly not *BRCA2* carriers (Anderson *et al*, 2003; Collaborators MWS, 2003; Rebbeck *et al*, 2005). Oestrogen-only HRT did not increase breast cancer risk in older women in the general population in the Women's Health Initiative (WHI) randomised trial (Anderson *et al*, 2003), and observational data from Denmark (Ewertz *et al*, 2005) indicates that it does not increase risk of breast cancer in women under 50 years. Based on these data, we and others offer HRT to carriers after surgery.

In this study, we investigated the uptake of HRT after BRRSPO and assessed menopausal symptoms and the use of, and results of, DXA scans used to monitor bone density in this population women.

## Materials and methods

### Study participants

Women were identified from our *BRCA1/2* database and a further database of patients at increased familial risk of ovarian cancer. Mutation carriers or other women with at least a 10% lifetime risk of ovarian cancer due to family history of ovarian ± breast cancer or Lynch syndrome and who had undergone BRRSPO were eligible. Women with BRRSPO >48 years were excluded, because of the potentially short period of oestrogen deprivation before a natural menopause. Women were sent a questionnaire and provided with pre-paid envelope for its return.

### Measures

The questionnaire enquired about ever or previous use of HRT, menopausal symptoms and whether DXA scans had been performed and their results.

The 18-item functional assessment of cancer therapy-endocrine symptoms (FACT-ES) questionnaire was used to assess menopausal symptoms. This has been shown to have acceptable validity and reliability for use in trials of endocrine therapy (Fallowfield *et al*, 1999). Women were asked to report occurrence of symptoms in the last 7 days, which were scored on a five-point Likert-type scale, ranging from ‘not at all’ to ‘very much’. These scores could then be reversed and summed to obtain a ‘total endocrine score’, ranging from 0 to 72, with lower values, indicating worse symptoms.

### Statistical analysis

Descriptive statistics were used to characterise basic demographic and medical information, and to determine HRT patterns of use and DXA scan information. The statistical package SPSSX version 16.0 (IBM Corporation, Armonk, NY, USA) was used for analysis. Although endocrine symptom data were negatively skewed, parametric tests were used to retain the score interpretability, as these are fairly robust on skewed data, between ‘never users’, ‘previous users’ and ‘current users’, and the answers to specific questions on the FACT-ES questionnaire and also between patients who were grouped into three time intervals for bone density comparison: ‘0’, ‘1–23’ and ‘⩾24 months’. In addition, Kruskal–Wallis and Mann–Whitney non-parametric tests were used for three and two group combinations to determine the relationship between the total FACT-ES score. All tests were two sided and *α*=0.05.

### Ethical approval

All research components of this study were performed with ethical approval of the Central Manchester Research Ethics committee.

### DXA scanning

Scans (DXA) were offered to all women who joined the study, although this was aimed primarily at those with bone unprotected by oestrogen prior to 50 years of age. The DXA was performed on a Hologic Discovery A DXA scanner with Apex System Software Version 2.3.2 software (Apex Systems, Hermitage, Berkshire, UK). Classification was undertaken using WHO criteria based on age-matched controls (WHO report, 1994). A T score in the lumbar vertebrae (L1–L4) or left neck of femur (hip) >−1.0 was considered normal; scores of −1.0 to −2.4 were considered as osteopenic and ⩽−2.5, osteoporotic.

## Results

### Demographics and medical information of participants

**Figure 1:**
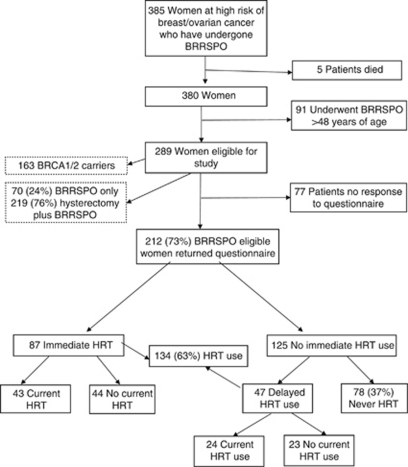
Flow diagram for BRRSPO cases.

We identified 385 women who had undergone BRRSPO from the Manchester Genetic Medicine Department Filemaker Pro databases. Five of these were found to have died at the time of the study, and 91 were excluded on the basis of age at oophorectomy. Two hundred and eighty-nine (163 from *BRCA1/2* mutation positive families) women were sent questionnaire packs, and 212 of these responded, giving a response rate of 73%. The patient characteristics of responders is provided in Table 1. Response rates among *BRCA1/2* families were a little higher: 123 out of 163 (75%) than for those from families not proven to be *BRCA1/2*: 89 out of 126 (71%). The median age of responders was 50 years (age range, 36–77). The median age at oophorectomy was 41 years (range, 24–48 years). Seventy-six per cent of women had hysterectomy at the time of BRRSPO, 24% had a BRRSPO only (Figure 1).

### HRT use

Women could be divided into never users of HRT (78 out of 212, 37%) and women who had taken HRT at some point (134 out of 212, 63%). In all, 87 women used HRT immediately after BRRSPO and 47 had a delay of up to 2 years before starting HRT. Of the patients receiving HRT, 67 out of 134 (50%) were current users at the time of completing the questionnaires and 67 (50%) were previous users (Figure 1; Tables 1 and 2). The majority used oestrogen-only preparations (79%). Only 7% used combination oestrogen and progesterone therapies, and 14% used other preparations, such as tibolone (*n*=12) and raloxifene (*n*=2). This use reflects that the majority of women had hysterectomy and BSO. The mean time of HRT use was 3.4 years (0.1–19 years). In all, 123 out of 212 (58%) women spent ⩾24 months before the age of 50 not taking HRT, resulting in a significant amount of time without oestrogen protection.

Most women (153 out of 212 (72%)) remember discussing the pros and cons of taking HRT with a health professional before treatment (8 were uncertain) and most (22 out of 37 (60%)) of those who did not discuss the issues wished that they had (12 did not answer the question). Women were asked why they chose not to use HRT and 56 out of 78 (72%) cited about breast cancer risk.

### Menopausal symptoms

Since the questionnaire was completed at different times after oophorectomy ranging from months to 36 years, and at differing ages, there were limitations to the interpretation of these scores. Women were divided into three groups for statistical analysis: those who had never used HRT (N), those who had previously used HRT but were not currently taking it (P) and those who were currently taking HRT (C). Of group (P), 44 out of 67 (66%) started HRT immediately after their BRRSPO. Results of covariate analysis were age when questionnaire completed (*P*=0.096), BRCA mutation (*P*=0.051) and HRT group (*P*=0.017). Six patients, two in each group, did not complete the FACT-ES forms. Although age did not appear to be a significant covariant, it was divided into 10-year groups (Table 2), a greater percentage of patients in the 40–49 age group had a total score <50 (the lower quartile of all groups), particularly in the past users (P) group.

Using independent sample *t*-tests to compare the means, there was a significant differences in total scores between all three groups analysed (N), (P) and (C) (*P*=0.017) This significant difference was largely explained by the difference between (P) and (C) (*P*=0.006) (Table 3). When split into 10-year age groups, the difference between these two HRT groups was largely explained by the age group 40–49 years (*P*=0.001). Current users (C) had a mean score of 58.7 compared with 53 for previous users (P). The 65 (P) had been off HRT for 0.2–11 (median 3.7) years, whereas never users were subject to oestrogen deprivation from BRRSPO for 0.5–29 years (median 5.2). The inter-group differences (N) (mean score 55.6) and (P) was neither significant (*P*=0.180) nor was it significant between (N) and (C) (*P*=0.093). There were no significant differences found when the other inter-group differences were compared.

In order to address a possible difference between women who delayed HRT use (D) and women who immediately took HRT (I), we assessed their total endocrine scores. There was no difference in total endocrine scores between group (I) (mean 56.7) and group (D) (median 55.5). Similarly, the proportions with scores of <50 between (I) and (D) was similar (26% 22%). The proportion of women who had never used HRT (N) with scores of <50 was 20%. Those questions, where at least 20% of all HRT groups answered ‘very much’ or ‘quite a bit’, are shown in Table 4 by HRT group. The percentage of patients currently on HRT treatment (C) experiencing ‘very much’ or ‘quite a bit’ appears to be less for hot flushes, night sweats and vaginal dryness.

Scores of <50 persisted up to 65 years of age and was generally more common in each age cohort among previous users (P) (Table 2).

### Bone protection

Our recently introduced guidelines indicate that after BRRSPO, women should have bone DXA scans every 2–5 years, if not taking HRT under 50 years of age. The mean period of non-HRT use among 139 women who were without oestrogen protection at some stage before age 50 was 5.2 years (range 1–19 years; median 5 years). One hundred and twenty-three women had at least 2 years of oestrogen deprivation before 50 years. In this retrospective study, only 73 out of 210 (36%) of women had a DEXA scan during an average period of risk <50 years of age of 6.33 years. According to the questionnaires, only 48 of 123 with ⩾24 months of oestrogen deprivation had undergone a DXA scan (40% scan findings were unavailable on six).

Scans have now been arranged on 119 women (Table 5). Of those who have now had a scan, 38% (45 out of 119) were found to have abnormal results. Based on the most recent DXA reports, 33 out of 119 (28%) reported reduced bone mass consistent with osteopenia and 12 out of 119 (10%) indicated osteoporosis. The prevalence of reduced bone mass was far higher among women who had ⩾24 months of oestrogen deprivation (36 out of 78 (46%)) than in those who had taken HRT to cover any period <50 years of age (5 out of 31 (16%)). The *χ*^2^ analysis of HRT groups and DXA results shows a significant difference between those with ⩾24 months of oestrogen deprivation and those with no deprivation <50 years (*P*=0.03). Of those who had not been scanned prior to the study who had ⩾24 months of oestrogen deprivation, 67 out of 71 (95%) wished to be scanned. However, 24 wished for their scans to be organised by their general practitioners and eight had not attended, thus results were only available on an additional 35 women.

## Discussion

To our knowledge, this is the only study to address the issues of HRT use, and both endocrine symptoms and bone health among women choosing BRRSPO because of an elevated risk of ovarian cancer. Madalinska *et al* (2005) did report on significant endocrine symptoms in women who had undergone BRRSPO for the same reason. In addition to the importance of adequate bone protection, BRRSPO puts women at risk of experiencing the symptoms of menopause from an early age. One way to combat both of these problems is to use HRT. This decision is not straightforward, however, especially in women with *BRCA1/2* mutations, because of the established links between HRT and breast cancer. Currently, there are no accepted guidelines on HRT use in this cohort of patients, although it has been suggested that it is acceptable for a short period of time in women who are experiencing symptoms (Armstrong *et al*, 2004; Rebbeck *et al*, 2005). Indeed, the reported lack of increase in breast cancer risk from HRT use after early BRRSPO (Rebbeck *et al*, 2005) suggests that the risk benefit ratio is in favour of use up until 50 years of age. Even a small abrogation of the protective effect of BRRSPO would be counterbalanced by the favourable effects of HRT on bone health and cardiovascular risks, mental health and the incidence of Parkinsonism when used at age <50 years (Anderson *et al*, 2003; Armstrong *et al*, 2004; Rivera *et al*, 2009; Rocca *et al*, 2009; Shuster *et al*, 2010) Once the immediate and long-term morbidity and mortality risks from bone density loss and heart disease are included, life expectancy is likely to be increased by HRT use up to 50 years of age (Armstrong *et al*, 2004; Rebbeck *et al*, 2005; Rivera *et al*, 2009; Salpeter *et al*, 2009; Howell and Evans, 2011).

We collected data on symptoms only at the time women filled in the questionnaire. Surprisingly, at this time, the women who were having the most severe symptoms (hot flushes, cold and night sweats and vaginal discharge) were previous users of HRT. This may be because of the knowledge of effectiveness while taking HRT. Never users would not have this experience of alleviation of symptoms and thus may perceive symptoms to be less severe. Our study suggests that for some women stopping HRT it may be difficult, since they are more aware of the benefits. In all, 65% of these women commenced HRT immediately, which means that only 35% would have initiated HRT use potentially due to bad menopausal symptoms. Importantly, this group who delayed commencement of HRT did not have significantly higher endocrine scores than those who started immediately after BRRSPO. Equally, the group who have never taken HRT may have had few symptoms and only those with the severest symptoms would choose to take HRT and thus end up in one of the other two groups. However, if this was the case, we might have expected worse endocrine symptoms among those women who had delayed HRT usage. There was no evidence of this with similar proportions of ‘immediate’ and ‘delayed’ use women having high endocrine scores and with similar mean and median scores. This was further supported by similar proportions of never users having severe symptoms to current users. This current study suggests that for some women taking HRT, it may more difficult to stop usage later, as they are aware of the benefits on their symptoms unlike those who have never taken HRT.

The risk of breast cancer in this cohort of women appears to be the main cause of concern about taking HRT. For many women, their decision making will have been before evidence that HRT does not appear to abrogate the protective effect of BRRSPO (Rebbeck *et al*, 2005), and the WHI randomised trial of oestrogen-only use showing reduction in breast cancer risk with oestrogen-only use, albeit in women aged 50–59 (Anderson *et al*, 2003; LaCroix *et al*, 2011). It is not clear why the largest observational trial, the Million Women Study, shows completely the opposite result in this age group (Beral *et al*, 2011) Uptake of BRRSPO in our centre is not as high as might be expected and the false fear of breast cancer risk being elevated against the concern about menopausal symptoms and HRT use may still be having an effect on uptake (Evans *et al*, 2009b). Our study showed that 77% of women stopped HRT due to worries about breast cancer risk. Counselling regarding the lack of evidence of breast cancer risk due to HRT in this cohort of patients is essential, in addition to highlighting the negative bone and cardiovascular effects of lack of use. We feel that the data available support the use of HRT in this population of women.

The *BRCA1/2* mutation carriers are recommended to undergo BRRSPO after the age of 35 years, or when childbearing is complete (Armstrong *et al*, 2004) In this retrospective study, we found that 59% (125 out of 212) women with early menopause due to BRRSPO chose not to have HRT after surgery, although 47 (22%) subsequently took HRT. Of those that did elect to take HRT at some stage, 134 women (63%) were on treatment for a mean of 3.4 years. A total of 133 women were without oestrogen protection at some stage <50, with 123 (58%) having at least 2 years oestrogen deprivation. Despite this, only 40% (48 out of 119) of women with significant oestrogen deprivation had been offered a DXA scan. Our study indicated that only 36% of women had ever had a DXA, and, of these, it was noted that 30% had osteopenia and 10% osteoporosis. Among women with ⩾24 months of oestrogen deprivation, 46% of women were osteopenic or osteoporotic. These data support those of Tham *et al* (2006), who reported that only 32% of women with chemotherapy-induced menopause had testing for BMD loss and for most this issue was not even discussed. Our study showed that most women (76%) wished to be scanned or to have their scans repeated and this rose to 95% of those with ⩾24 months of oestrogen deprivation. Currently, no single health-care provider appears to be taking control of bone health in this group of women. Hormone replacement therapy may be started by the gynaecologist, but later the woman or her general practitioner may stop HRT before the natural menopause age (∼50 years). It is important, therefore, to not only impress upon health-care providers the importance of DXA scanning in this group of patients, but to ask them to discuss it with women and relate to them current guidelines. Data indicate that the greatest proportion of BMD loss occurs in the first 18 months following surgery, after which the rate of loss begins to decrease (Cann *et al*, 1980). It may, therefore, be useful to amend the current guidelines to assess BMD at the time of surgery or before surgery for a baseline measurement, scan again at 1 or 2 years to detect any rapid BMD loss that may need treatment, and then again at 2 years before switching to every 5 years once the rate of loss has slowed.

This study has several limitations, including the retrospective nature. We relied on the woman's own memory of HRT usage and there are likely to be errors regarding duration of HRT. We do not know the reasons for starting/stopping medication. However, it is likely that women's knowledge of never or continual use is accurate. The response rate to the questionnaire of 73% is good, but it is possible that women with greater concerns about bone health may have responded disproportionately. Clearly, a prospective study would inform further regarding issues of bone and other health issues in women undergoing early BRRSPO.

We have demonstrated that endocrine symptoms are significantly more severe in previous users of HRT compared with current users and there is a high rate of bone loss in women who have ⩾24 months of oestrogen deprivation prior to 50 years of age. Thus women should be encouraged to take continuous HRT for better control of symptoms after BRRSPO at least until they reach 50 years and counselled concerning the pros and cons of this advice (Howell and Evans, 2011).

## Figures and Tables

**Table 1:** Patient characteristics of 212 women returning questionnaires

	**Immediate HRT (I)**	**Delayed HRT (D)**	**No HRT (N)**	**Total**
Number	87 (%)	47 (%)	78 (%)	212 (%)
Age at surgery median (range)	41.7 (24–48)	40.4 (27.5–48)	41.4 (31–48)	41.2 (24–48)
*BRCA1/2* family	44 (51)	30 (64)	49 (63)	123 (58)
Family history of ovary/breast cancer	42 (49)	17 (46)	29 (37)	88 (42)
Current users	43 (50)	24 (46)	—	67 (32)
Previous users	44 (51)	23 (48)	—	67 (32)
⩾24 months no HRT <50 years	27 (31)	24 (51)	72	123 (58)

Abbreviation: HRT=hormone replacement therapy.

**Table 2:** Age at current assessment and substantial endocrine symptoms by FACT-ES in 206 women returning questionnaires

**Age at assessment**	***N***	**HRT group**	***n***	**Score<50**
30–39	12	N	4 (33%)	1 (25%)
		P	3 (25%)	—
		C	5 (42%)	1 (20%)
40–49	95	N	33 (35%)	9 (27%)
		P	22 (23%)	11 (50%)
		C	40 (42%)	6 (15%)
50–59	77	N	31 (40%)	7 (23%)
		P	28 (36%)	8 (29%)
		C	18 (24%)	5 (28%)
60+	22	N	8 (36%)	3 (38%)
		P	12 (55%)	1 (8%)
		C	2 (9%)	—

Abbreviations: C=current users; FACT-ES=functional assessment of cancer therapy-endocrine symptoms; HRT=hormone replacement therapy; N=never users; P=past users.

**Table 3:** Comparison of total endocrine scores between HRT groups

**Length of oestrogen deprivation**	***N* a**	**Mean score^b^**	**s.d.**	***t*-value**	**DF**	***P*-value**
N *vs* P *vs* C	76	55.6	10.8	4.18	205	0.017
	65	53.0	12.0			
	65	58.7	11.0			
N *vs* P				1.35	139	0.180
N *vs* C				−1.69	139	0.093
P *vs* C				−2.82	128	0.006
						
*Age group 40–49 years*						
N *vs* P *vs* C	33	56.2	10.5	6.25	94	0.003
	22	48.9	10.2			
	40	59.0	11.4			
P *vs* C				−3.46	60	0.001

Abbreviations: C=current users; DF=degrees of freedom; FACT-ES=functional assessment of cancer therapy-endocrine symptoms; HRT=hormone replacement therapy; N=never users; P=past users.

a Two from each group did not complete FACT-ES form.

b Scores are reversed, that is, max 72 equates to no symptoms.

**Table 4:** Individual questions where ⩾20% of all patients experienced a symptom ‘very much’ or ‘quite a bit’

**Endocrine symptom**	**Experiencing ‘very much’ or ‘quite a bit’ (%)**			
**HRT group**	**All**	**N**	**P**	**C**
Lost interest in sex	34	39	30	31
Gained weight	32	28	39	31
Hot flushes	24	25	32	15
Night sweats	23	26	33	9
Vaginal dryness	23	25	31	14
Feel bloated	20	13	28	22

Abbreviations: C=current users; HRT=hormone replacement therapy; N=never users; P=past users.

**Table 5:** Prevalence of osteopenia and osteoporosis in 119 women undergoing risk-reducing oophorectomy aged 48 years or younger

**Length of oestrogen deprivation**	**Median age at BRRSPO (range)**	**Median age at DXA (range)**	**DXA normal**	**Osteopenia (DXA T score −1.0 to −2.4)**	**Osteoporosis (DXA T score <−2.4)**
0	42.6 (31–48)	49 (41–61)	26 (84%)	4 (13%)	1 (3%)
1–23 months	42.9 (34–48)	50 (32–68)	6 (60%)	3 (30%)	1 (10%)
⩾24 months	41.1 (24.9–48)	50 (38–78)	42 (54%)	26 (33%)	10 (13%)

Abbreviation: BRRSPO=bilateral risk-reducing salpingo-oophorectomy.

Difference between no oestrogen deprivation group and ⩾24 months group significant for reduced bone density (osteopenia or osteoporosis), *P*=0.03.
